# Physician-guided, hybrid genetic testing exerts promising effects on health-related behavior without compromising quality of life

**DOI:** 10.1038/s41598-021-87821-8

**Published:** 2021-04-19

**Authors:** Severin Schricker, David Callau Monje, Juergen Dippon, Martin Kimmel, Mark Dominik Alscher, Moritz Schanz

**Affiliations:** 1 Department of General Internal Medicine and Nephrology, Robert-Bosch-Hospital, Auerbachstraße 110, 70376 Stuttgart, Germany; 2 Department of Mathematics, University of Stuttgart, Pfaffenwaldring 57, 70569 Stuttgart, Germany; 3 Department of Internal Medicine, Division of Nephrology, Hypertension and Autoimmune Disorders, Alb-Fils Kliniken, Eichertstraße 3, 73035 Göppingen, Germany

**Keywords:** Genetics research, Clinical trial design

## Abstract

Genetic risk analysis is increasingly in demand by participants. Hybrid genetic testing has the advantage over direct to consumer testing by involving a physician who guides the process and offers counseling after receiving the results. The objective of this study was to determine whether a structured physician moderated primary preventive, hybrid genetic risk assessment enhanced counseling program leads to improvement in lifestyle and does not impair quality of life. Risk genes for malignant, cardiovascular, coagulation, storage diseases and pharmacogenetics (> 100 genes) were tested. Screening, consultation and genetic counseling embedded in a primary/secondary prevention check-up program for executives of surrounding companies took place in a single center in Germany. Follow-up included established questionnaires for quality of life, nutrition and physical activity. Analysis included n = 244 participants. Median age at baseline was 49 years (interquartile range: 44–55), 93% were male, 3% (n = 7 of 136 responses) were smoker. Mean body mass index was 25.2 kg/m^2^. Follow-up response rate was 74% (n = 180), mean follow-up time was 6.8 months (standard deviation = 2.1). In 91 participants (37.8%, 91/241) at least one pathogenic variant was found, 60 thereof were clinically relevant (24.9%, 60/241). 238 participants (98%, 238/241) had > 1 pharmacogenetic variant, only 2 (0.8%, 2/241) took a correspondingly affected drug (56 participants took ≥ 1 drug/day). The energy expenditure significantly increased by ≈ 35% [median multiple of energy expenditure of 1.34 (confidence interval = 1.15–1.57, p < 0.001)] metabolic equivalents of task (MET)-min/week; participants spent on average 41 min (p < 0.001) less in sedentary activities per day and spent more time for lunch (≈ 2 additional minutes/day; p = 0.031). Indicators of the consumption of red meat and sweet pastries significantly decreased (both adjusted p = 0.049). Neither quality of life in general nor subgroup analysis of participants with at least one conspicuous genetic risk differed significantly over follow-up. Hybrid genetic testing and counseling exerted positive effects on health-related behavior and was not associated with major psychological adverse effects in the short-term follow-up. The approach seems to be feasible for use in preventive health care.

## Introduction

Genetic panel diagnostic enables the possibility of creating a risk profile and derive individual preventive measures^1–3^ but the value depends on appropriate behavioral changes of participants and measures taken by treating physicians in response to increased risk, leading to prevention and early detection of disease^1,4^. For a long time, genetic testing was thereby the preserve of human geneticists, who initiated targeted and occasion-related diagnostics and assumed central responsibility for the tests, such as ordering the tests and communicating the results. In recent years, as sequencers and panel diagnostic modules became more affordable, so-called direct-to-consumer (DTC) models were established, whereby any medical layperson could have genetic screening tests performed without the involvement of a physician, sparking controversy about adverse effects^1,5,6^. One newer approach is to involve a physician in moderating third party broad genetic panel diagnostics while maintaining consumer involvement. A physician moderates the decision for testing, orders the test and returns the results. This was recently described as hybrid genetic testing (hGT)^3^ as a middle ground between the DTC model and the traditional model, combining benefits from both models while excluding potential negative aspects. This approach has the distinct advantage that, unlike the DTC model, results translate directly into clinical decision-making and protects consumers from being left alone with their results, unlike DTC testing.

However, research on the effects of hybrid physician-guided testing and counseling is lacking. Therefore, the objective of this study was to determine whether a structured physician-moderated preventive, hybrid genetic risk assessment enhanced counseling program exerts positive effects on health-related behavior without compromising quality of life.

## Subjects and methods

### Trial design and participants

This study recruited participants from outpatient preventive medicine check-up visits from April 2016 to December 2017 at the Robert-Bosch-Hospital, a 1,041-bed referral center in Stuttgart, Germany.

A preemptive genetic panel diagnostic analysis for modifiable genetic risk factors was performed as part of a primary/secondary preventive medical care approach mainly for executives of leading industrial companies in our locality as well as self-paying patients. As part of the preventive medical care program various screening examinations (detailed medical history, physical examination, ultrasound, X-ray, and laboratory tests) were carried out. Every patient enrolled in this preventive medical care program and interested in genetic testing was asked to participate in this study.

The genetic panel is commercially obtainable and processed by CeGaT (Tübingen, Germany). It includes seven “modules” on risk variants regarding malignancies (e.g., BReast CAncer (BCRA) 1/2), cardiovascular diseases, coagulation (e.g., thrombophilia), cholesterol and storage disorders (e.g., hemochromatosis), glaucoma, and pharmacogenomic details (> 100 genes, see Supplementary Table S1).

**Figure 1:**
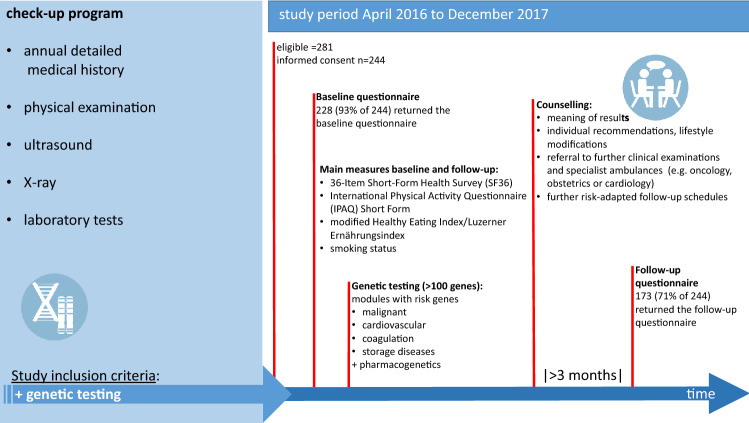
Study setting and participant flow chart.

After receipt of the genetic test results, all participants received detailed clarifications and counseling by the same senior specialist in the field of internal medicine and human genetic counseling. The individual recommendations regarding meaningful lifestyle modifications, and the referral to further clinical examinations in specialist ambulances (e.g. oncology, obstetrics or cardiology) or further risk-adapted follow-up schedules were provided by the physician who indicated the tests and treated the participants (as stated by German law). In addition, the participants received a detailed written report by CeGaT including the tested genes, relevant identified variants, and a summary of clinical importance, supporting evidence, and the offer of genetic counseling provided by a specialist in human genetics provided by the physician network of CeGaT. We summarized the process in (Fig. 1).

### Ethics and consent

This study was conducted after consultation with the ethics committee of the University of Tubingen, Germany (Process Number: 197/2016BO1). All research was performed in accordance with relevant guidelines and all participants provided written informed consent.

#### Inclusion/exclusion criteria

The study included participants aged > 18 years who agreed to the genetic testing and to participate in the study. Preplanned exclusion criteria were: (1) for liability reasons: age < 18 years and presence of pregnancy (possible adverse effects of genetic risk results), (2) In order to exclude a proportion of participants with significantly reduced life expectancy and quality of life: requirement for dialysis and active cancer, and (3) Formally: no or revoked informed consent for genetic testing, no or revoked informed consent to participate in the study.

#### Outcome measurements and data collection

The genetic test results were interpreted by the investigators: The term “not clinically significant” was defined as not meaningful for the prevention of disease or health of the individual (e.g., in the case of heterozygote alleles in recessive hereditary risk factors or diseases, or genes lacking clinically meaningful data based on current evidence).

Demographic and clinical data (i.e., age, height, weight, and blood pressure), family history, (drug) anamnesis, and diagnoses were obtained at baseline (before receiving the genetic test results) from the clinical source data.

The participants were assessed at two time points by questionnaires: at baseline and in a follow-up (≥ 3 months after return of the genetic test results). The baseline survey was handed out and conducted on site at presentation directly after inclusion in the study. The follow-up questionnaires, on the other hand, were sent and also returned to us by mail. A complete baseline assessment was not mandatory to be further included in analysis. Both baseline and follow-up questionnaires included the following questions and well-established instruments:

##### 36-item short-form health survey (SF36)

The generic norm-based 36-Item Short-Form Health Survey (SF36) was used to assess participants’ perceived quality of physical and mental health. The physical (SF36-psc) and mental (SF36-msc) subscales’ scores are computed by weighted averaging of the single items of the questionnaire following the manual of the test and the “general current and chronic disease” normalization of the first version Bullinger and Kirchberger^7^. Components with lower scores thereby resemble lower levels of perceived health.

##### International physical activity questionnaire (IPAQ) short form

We employed the short version of the instrument with seven items to be completed by the respondent. The IPAQ is an instrument for monitoring of physical activity and inactivity behavoir of a normal week^8^. The IPAQ items are computed into physical activity measured as metabolic equivalents (MET, higher values indicate more physical activity) per week and time spent in hours in a sedentary position.

##### Modified healthy eating index/luzerner ernährungsindex (LEI: Lucerne Nutrition Index, eating habits at lunchtime)

We employed the lunchtime assessment of the LEI^9^, a instrument in german based on the American healthy eating index (HEI)^10^. This questionnaire asks about various components, accompanying beverages, the setting, and the location of the midday meal according to their frequency on a Likert scale from 1 "never" to 4 "almost daily" and 0 "I don't know," respectively. Additionally, time spent on the lunch is reported.

##### Additional questionnaires

We observed smoking status by asking: “Do you smoke?” (“Yes”/”No”) and “if yes, how many cigarettes do you smoke per day?”.

Follow-up questionnaires included following additional questions: “Have you implemented the recommendations with regard to your lifestyle?” (“Yes”/“No”/“No measures were necessary”, comment field for specifications) and “Have you subjectively changed your eating habits compared to before the test?” (“Yes”/“No”/“No measures were necessary”, comment field for specifications). Moreover, a Likert scale (from 0 to 10) was used to assess the satisfaction with genetic screening and counseling.

### Statistical analysis and data handling

Predefined primary endpoints were: (1) Influence of genetic screening on quality of life, recorded by SF36 questionnaire, and (2) Influence of genetic screening on lifestyle, recorded by HEI/LEI and IPAQ questionnaires. Secondary endpoints were participant satisfaction with genetic screening, influence of genetic screening on smoking habits, on quality of life and lifestyle in the subgroups “at least 1 positive result in modules 1–6” and “unremarkable results in modules 1–6”.

The investigators had access to the database and were involved in the clinical treatment of the eligible study population. Patients for whom consent could not be obtained were not covered by the ethics vote and therefore not systematically included. Study nurses and the investigators transcribed data from the clinical information system and questionnaires of participants into one database. We did not employ any database linkage or methods for the imputation of missing values in this study. The physical (SF36psc) and mental (SF36msc) subscale scores were computed by weighted averaging of the single questionnaire items, employing IBM SPSS Statistics for Windows, Version 20.0 (Released 2011. Armonk, NY: IBM Corp) and the tool for normalization provided by the manufacturer (Hogrefe, Göttingen, Germany).

For each item in the questionnaires, differences in paired observations (follow-up minus baseline values) were assessed by multivariable linear regression. As age, gender, and BMI possibly influenced the outcome, these quantities were included in the model after centering at their respective mean. This allows taking the intercept as an effect measure and testing the hypothesis of no effect. Since baseline and follow-up distributions of MET-min (IPAQ measure of physical activity) were highly skewed, both variables were logarithmized. Exponentiation of the intercept hereby results in an estimate of the median ratio of follow-up and baseline values. Because non-participation in follow-up positively correlated with age, follow-up data were not missing completely at random (MCAR), but we believe that the missing at random (MAR) assumption is nevertheless acceptable. All p-values refer to two-sided alternatives. Regarding all items of the LEI score, p-values were adjusted for multiple testing to control for the false discovery rate (FDR, named adjusted p)^11^. The inferential analyses were performed using the statistical computing language R^12^, version 4.0. Percentages were rounded to whole numbers.

## Results

In this study 281 consecutive and unselected check-up patients, who were interested in hybrid genetic risk testing, were eligible and approached for participation. A total of 244 participants provided informed consent prior to the initiation of the genetic testing and were included in the analysis.

Descriptive statistical data and the demographic information of the participants with available data are presented in Table 1.

**Table 1:** Subject characteristics.

Characteristics	Unit	Value	Number of missing values
Number of subjects (%)	n	244 (100%)	–
Age (median, IQR)	years	49 (44–55)	0
Women (%)	n	18 (7%)	0
Body height (median, IQR)	cm	182 (178–1867)	17
Body weight (median, IQR)	kg	84 (77.0–90.5)	17
Body mass index (mean, 95%CI)	kg/m^2^	25.2 (23.5–27.2)	31
Systolic blood pressure (median, IQR)	mmHg	140 (125.0–150.0)	16
Diastolic blood pressure (median, IQR)	mmHg	80 (75.0–80.0)	16
Peripheral oxygen saturation, SpO_2_ (median, IQR)	%	95 (93–96)	17
Smoker (%)	n	7 (3%)	108
Number of daily drugs per participant, (median, range)	n	1 (1–5)	3

*cm* centimeter, *IQR* Interquartile range, *kg* kilogram, *mmHg* unit millimeter of mercury, *NA* number of missing values, % percent.

The cohort included young (range 30–74, 98% < 65 years), mostly male subjects. Of the 244 participants included in the analysis, 228 (93% of 244) returned the baseline questionnaire and 173 returned the follow-up assessment (71% of 244). Subjects returned the follow-up questionnaires after 6.8 months on average (standard deviation = 2.1) when receiving their results of the genetic test.

### Genetic test results

The results of the genetic test were available for 241 (99% of 244) participants. Of those, 91 (37.8% of 241) exhibited positive findings (without module 7/pharmacogenomic analysis). Sixty individuals showed at least one finding considered as clinically significant (24.9%, 60 of 241). Clinically relevant risk gene variants were mainly identified for thrombophilia and malignancies (see details in Supplementary Table S2).

Only 56 participants (24% of 241) took any and most of them only one daily medication (in median 1, range 1–5 drugs per day). While almost all participants (98%, 238 of 241) had at least one pharmacogenetic variant that could affect future prescriptions, only two participants took a correspondingly affected medication during the course of the study (both CYP2C19 ultra-rapid metabolizers who took pantoprazole).

### Outcome measures

Overall satisfaction with the genetic testing and counseling was high (Likert scale median = 9). Of the participants who reported about their compliance with advised measures in follow-up (n = 170, 70% response rate), the majority (58%, 98 of 170) did receive a recommendation for a lifestyle change. Of those, 54% (n = 53) subjectively did not adopt the advised measures until follow-up assessment.

**Figure 2:**
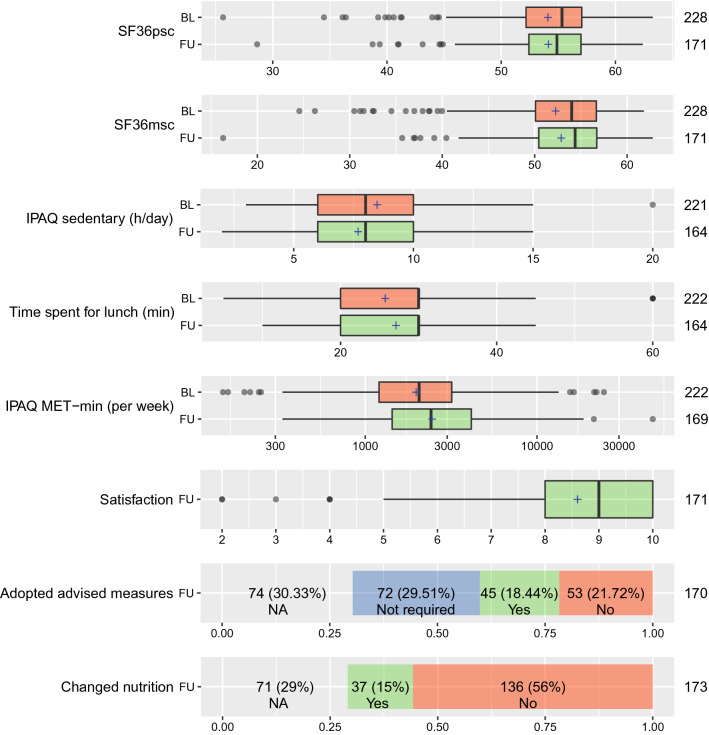
Outcome measures at baseline and follow-up in the whole cohort. *Dots* values of outliers, *whiskers* 95% interval, *box* interquartile range, *line within box* median value, cross within box = arithmetic mean, nominal data in bars: number (percent of all participants). *BL* baseline, *FU* follow-up, *n* number, *min* minute, *h* hour, *IPAQ* International Physical Activity Questionnaire, *MET-min* metabolic equivalent of task per minute, *NA* number of missing values, *SF36msc* mental health subscale of the Short-Form Health Survey, *SF36psc* physical health subscale of the Short-Form Health Survey. Graph was created with R^12^.

Indicators of quality of life (SF36 mental and physical subscales) did not differ significantly between follow-up and baseline in the whole cohort. Overall, we observed positive changes in the measures of health-related behavior, physical activity (PA), and nutrition (see section below). The preventive medical care counseling did not result in the cessation of smoking among the seven smokers included in our cohort. The results of the descriptive statistical analysis for the outcome variables of interest are presented in Fig. 2.

**Figure 3:**
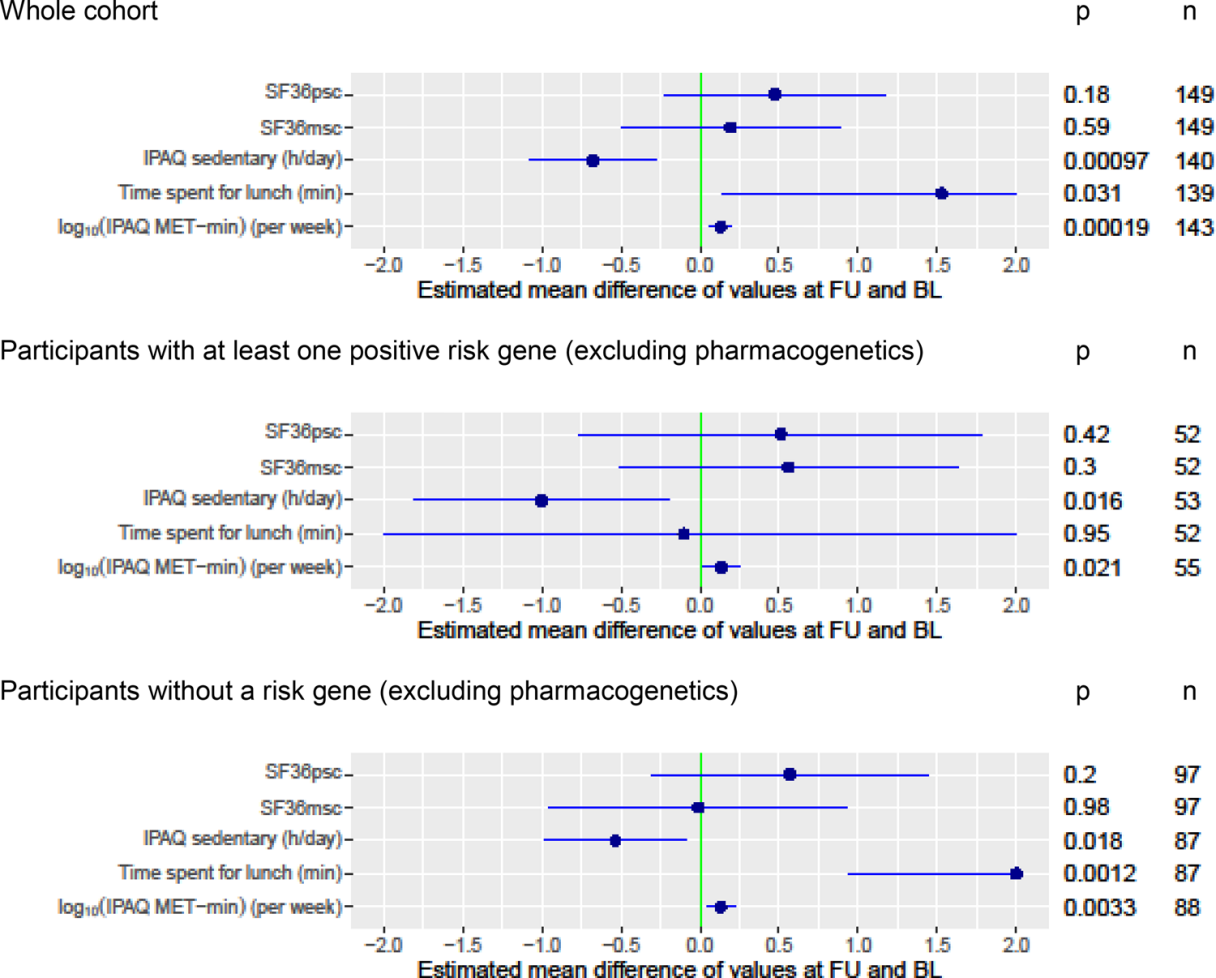
Subgroup analysis of differences in outcome measures between follow-up and baseline. Dots = estimated mean difference after adjusting for age, gender, and body mass index, whiskers = 95% confidence interval, clipped when exceeding the displayed range, X-axis: the estimated mean difference between follow-up and baseline (FU-BL), except for IPAQ MET-min, where we consider the difference of logarithmized values. *BL* baseline, *FU* follow-up, *h* hour, *IPAQ* International Physical Activity Questionnaire, *MET-min* metabolic equivalent of task per minute, *min* minute, *n* number, *p* raw p-values, *SF36msc* mental health subscale of the Short-Form Health Survey, *SF36psc* physical health subscale of the Short-Form Health Survey. Graph was created with R^12^.

We found significant differences in energy expenditure and sedentary behavior between baseline and follow-up. The energy expenditure significantly increased by ≈ 35% (median multiple of energy expenditure of 1.34, confidence interval = 1.15–1.57, p < 0.001) measured in metabolic equivalents of task (MET)-min/week. In addition, participants spent on average 0.68 h (≈ 41 min, estimated mean difference, p < 0.001) less in sedentary activities per day (see also Fig. 3).

#### Subgroup analysis for genetic risk

Subgroup analysis with an unremarkable genetic test result (excluding pharmacogenetics) and participants with at least one clinically relevant finding did not reveal significant differences (see Fig. 3). Time spent sedentary decreased more pronounced in the group with positive findings, PA increased similarly in both groups. Results of the subgroup analysis per genetic risk module are presented in Supplementary Fig. S1. Overall, those subgroups per module were small. Therefore, we could not find any noteworthy estimated mean differences of outcome measures within single modules on the outcome measures.

#### Changes in nutrition

**Figure 4:**
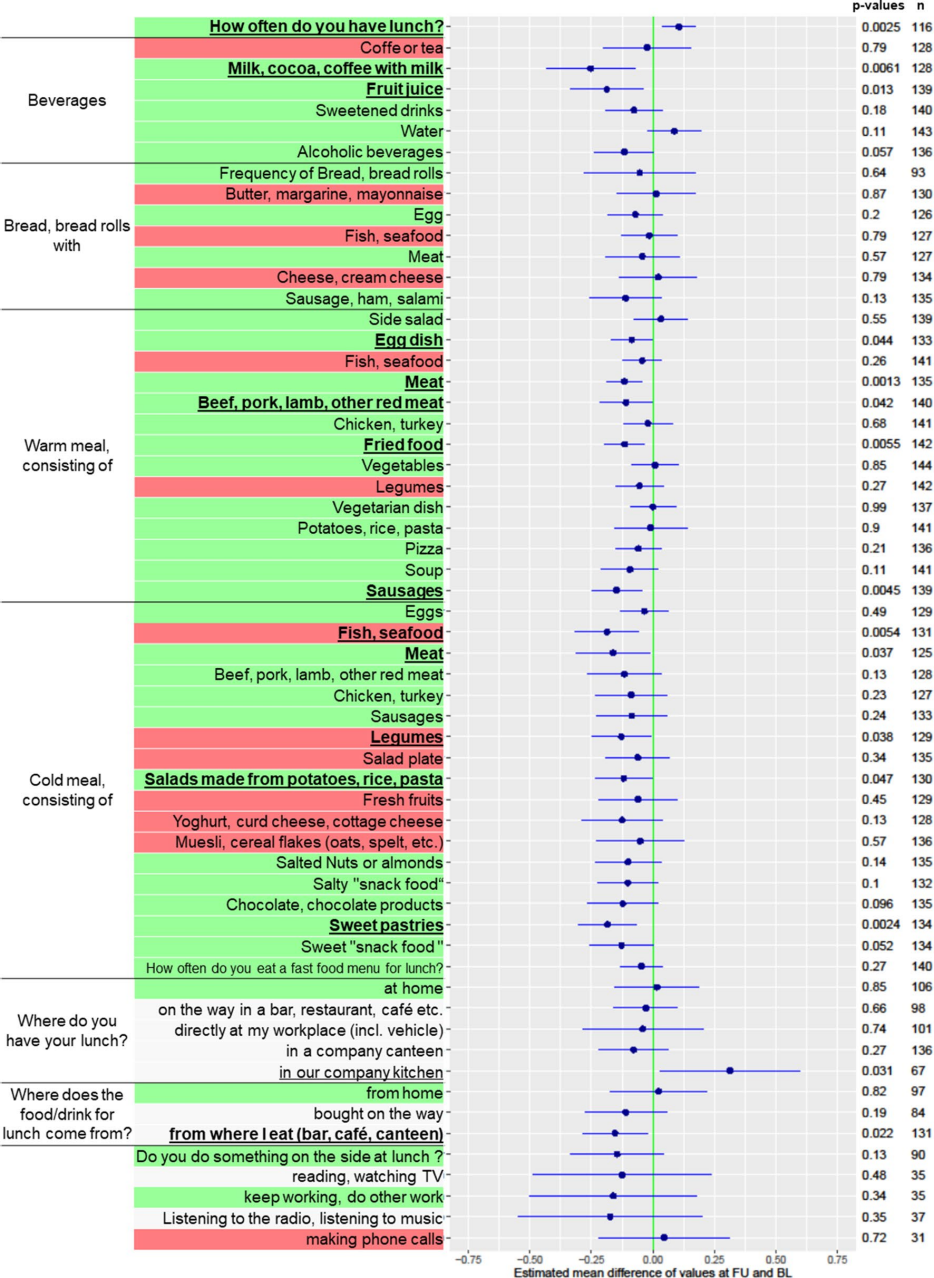
Change in nutrition (at lunchtime) per questionnaire item of the LEI score for the entire cohort. Raw p-values in columns, items underlined and bold when < 0.05, *n* = number of cases, highlighted green = difference towards direction of desired behavioral changes, highlighted red = difference towards direction of undesired behavioral change, highlighted gray = no rating, indifferent. Graph was created with R^12^.

In follow-up, the majority of participants (77%, 136 of 173 who answered the question) reported that they subjectively did not change their eating habits. Within the questionnaires, participants had significantly more often lunch (adjusted p = 0.049) and spent an increased amount of time at lunch (estimated mean difference ≈ 2 min per day additionally; p = 0.031). Of note, the variance of the duration of the lunch was markedly higher in the subgroup with positive findings, whereas the subgroup without finding showed a significant increase of ≈ 2 min per day additionally. The changes of single questionnaire item scores regarding eating habits at lunchtime (i.e., LEI) mainly directed towards behaviors that are more preferable (39 of 51 rated items ≈ 76%, for definitions see Supplementary Table S3). Indicators of the consumption of red meat (adjusted p = 0.049) and sweet pastries (adjusted p = 0.049) significantly decreased (see Fig. 4).

That is coherent with 45 qualitative commentaries provided by the participants (see Supplementary Table S4). In addition, we compared the nutritional behavior of the study participants with an inconspicuous genetic test result (excluding pharmacogenetics) with participants with at least one positive result (see Supplemental Fig. S2). Here we found no significantly different changes in diet. Thus, the change in dietary behavior of the participants seems independent of the genetic test. Of note, a selected and most pronounced exception was the consumption of insignificantly more bread rolls, beef, and fresh fruit in participants with positive risk genes.

## Discussion

Genetic testing may be helpful in identifying participants at risk for certain diseases and personalizing preventive measures to maintain and improve health and quality of life^2,13^. The objective of this study was to analyze the effects of hybrid genetic testing in a primary/secondary preventive check-up program on health-related behavioral change, well-being, and satisfaction with counseling. According to our study genetic panel screening does not appear to have adverse effects on the psychological well-being or quality of life of participants. Moreover, this approach exerts promising effects on health-related behavior, nutrition, and physical activity. In addition, we showed that subjective participant satisfaction with genetic testing was high.

Studies on genetic panel diagnostic so far primarily concentrated on effects of direct-to-consumer (DTC) tests^13^, which have repeatedly led to controversy: although, the results indicated some positive effects^14,15^, several studies have even reported unhealthy behavioral responses to low risks of disease^13^, thwarting the main mechanism of disease prevention. Furthermore, genetic information and risk are difficult to interpret^16,17^. There is evidence indicating that DTC testing may increase healthcare usage^18,19^ resulting in additional burden to the healthcare system and primary physicians^20^. In part, these effects can be attributed to a setting lacking the involvement of a guiding physician—an approach we employed in this study.

Our results regarding effects on quality of life are in line with several studies in the field—there has not been reported any convincing evidence of adverse psychological effects so far^13,15,21–24^.

Of note, other relevant studies reported that their examined populations were mostly free of high-risk genes that may facilitate the occurrence of adverse effects^20^. In the present study, we observed a reasonable frequency of pathogenic variants to draw conclusions regarding the impact of genetic risk findings in general. Since subgroups of risk modules (e.g. for malignancies) or even single genes were nevertheless small, we were unable to show a specific impact of single modules on outcome measures.

In our model, we found generally positive changes in health related behavior in the whole cohort (increased PA, healthier composition of nutrition and lunchtime habits (increased time spent on lunch, also see qualitative statements in Supplementary Table S4). Thus far, evidence shows mixed results in terms of triggered behavioral changes and suggest that genetic risk assessment and counselling has little effect on risk-reducing health behavior (as reviewed in^25^). A number of observational studies employing direct-to-consumer tests found positive indicators^20,26^, whereas others did not identify associations with diet or exercise^15,21,23^. This is also true for newer randomized, controlled studies, which showed on the one hand positive changes in dietary behavior^27^ and on the other hand, no significant changes in objectively measured physical activity following personalized advice based on genetic risk^28^. In our study, we also only archived compliance in around 50% of the cohort. These observations emphasize the need for improved educational strategies to encourage lifestyle changes^29,30^. Therefore, genetic testing, focusing on improved participant education and decision-making, should be proposed along with proper psychological support, counseling and further medical care. To the best of our knowledge, there are no published randomized, controlled studies investigating effects of real-life hybrid genetic testing employing large genetic risk panels on lifestyle and hard endpoints as mortality or morbidity.

Of note, in the study presented here almost all participants had a potentially clinically relevant finding within the pharmacogenomics panel, but the results had an impact on only two participants with relevant medication. This underlines the potential of integrating genetic screening into primary care, as virtually all participants are likely to benefit in the future with an increasing number of prescribed drugs. Future studies should therefore integrate a longtime follow-up, to observe potential benefits and harm in the long term– being the very point of a primary preventative approach.

## Limitations

Our cohort consisted mostly of males in relatively good health, with an already healthy behavior. This homogeneity and our design as a single-center study leads to a decreased external validity. However, we see a chance to present the possibilities and effects of a primary preventative approach. By eliminating possible confounders on our endpoints (e.g. quality of life and lifestyle), examining a very homogeneous collective, embedding genetic testing and counseling in a maximally uniform setting, as well as standardizing a diagnostics schedule beforehand may pose as a proof-of-principle. Nevertheless, we cannot exclude that other social groups that lack these attributes may experience other outcomes following genetic testing. As we could not collect information about patients who refused to participate in the study, we were not able to address possible systematic differences with those patients who agreed. Therefore, generalizability of our findings needs further research. Further, the sample size of this study was reasonably large, but it did not include the large numbers of participants that were included in studies investigating the effects of DTC^13^. Nevertheless, to the best of our knowledge, we present the largest study integrating hybrid genetic testing in a preventive strategy.

## Conclusion

Hybrid genetic testing and counseling exerted positive effects on health-related behavior and was not associated with major psychological adverse effects in the short-term follow-up. The approach seems to be feasible for use in preventive health care. Investigations including long-term follow-ups are warranted to determine the sustainability of positive effects, and whether these effects are translatable into improved hard endpoints (i.e., mortality and other health-related outcomes).

## Supplementary Information

Supplementary Information 1.

Supplementary Figure 1.

Supplementary Figure 2.

## Data Availability

De-identified participant data (including data dictionaries), used questionnaires, study protocol, statistical analysis plan, etc. will be shared upon request and after review of institutional policies on data protection.
