# Supplementary material for: Physician-guided, hybrid genetic testing exerts promising effects on health-related behavior without compromising quality of life
Source: Sci Rep. 2021 Apr 19;11:8494. doi: 10.1038/s41598-021-87821-8 (PMC8055666; doi:10.1038/s41598-021-87821-8)
Supplement: Supplementary file 1 — Supplementary Information 1. [file 41598_2021_87821_MOESM1_ESM.docx]

**Physician-guided, hybrid genetic testing exerts promising effects on health-related behavior without compromising quality of life**

Short title: Impact of pre-emptive hybrid genetic testing

Severin Schricker^1^, David Callau Monje^1^, Juergen Dippon^2^, Martin Kimmel^3^, Mark Dominik Alscher^1^, Moritz Schanz^1^

^1^ Department of General Internal Medicine and Nephrology, Robert-Bosch-Hospital, Auerbachstraße 110, 70376 Stuttgart, Germany

^2^ Department of Mathematics, University of Stuttgart, Pfaffenwaldring 57, 70569 Stuttgart, Germany

^3^ Department of Internal Medicine, Division of Nephrology, Hypertension and Autoimmune Disorders, Alb-Fils Kliniken, Eichertstraße 3, 73035 Göppingen, Germany

| **Supplementary Table 1. Detailed list of genetic variants tested.** | | |
| --- | --- | --- |
| **Module name** | **Disease of interest** | **Genecard identifier** |
| **Module 1: Malignancies** | Colon carcinoma; breast and ovarian carcinoma; prostate carcinoma; pheochromocytomas and paragangliomas; familial tumor syndromes; pancreatic carcinoma; renal cell carcinoma; familial melanoma; RASopathies.  >100 gene variants | A2ML1, AKT1, AKT3, APC , AR, ATM, ATR, Axin2, BAP1, BLM, BMPR1A , BRAF, BRCA1, BRCA2, BTNL2, CBL, CCND2, CD82 , CDC73, CDH1, CDK4, CDKN1B, CDKN2A, CHEK2, CYLD, DICER1, ELAC2 , EPCAM , FH, FLCN, HNF1A, HNF1B, HOXB13, HRAS, KRAS, LIG4, LZTR1, MAP2K1, MAP2K2, MAX, MC1R, MEN1, MET, MITF, MLH1 , MSH2 , MSH6 , MSR1 , MUTYH , MXI1 , NBN, NF1, NF2, NRAS, PALB2, PALLD, PIK3CA, PIK3R2, PMS2 , POLD1, POLE, POT1, PRKAR1A, PRSS1, PTEN, PTPN11, RAD51C, RAD51D, RAF1, RASA1, RASA2, RASAL1, RB1, RECQL4, RET, RINT1, RIT1, RNASEL , RRAS, SDHA, SDHAF2, SDHB, SDHC, SDHD, SHOC2, SMAD4, SMARCA4, SMARCB1, SMARCE1, SOS1, SPINK1, SPRED1, STAMBP, STK11, TMEM127, TP53, TSC1, TSC2, VHL, WRN, WT1, ZFHX31 |
| **Module 2: Cardiology** | Cardiomyopathies, cardiac arrhythmias, aneurysms  >90 gene variants | ABCC9, ACTC1, ACTN2, ACVRL2, AKAP9, ANK2, ANKRD1, BAG3, CACNA1C, CACNA2D1, CACNB2, CALM1, CALM2, CALR3, CASQ2, CAV3, CRYAB, CSRP3, CTNNA3, DES, DMD, DPP6, DSC2, DSG2, DSP, DTNA, EMD, ENG, EYA4, FHL1, FKTN, GATAD1, GDF2, GJA5, GLA, GNAI2, GPD1L, HCN4, JPH2, JUP, KCNA5, KCND3, KCNE1, KCNE2, KCNE3, KCNH2, KCNJ2, KCNJ5, KCNJ8, KCNQ1, LAMA4, LAMP2, LDB3, LMNA, MIB1, MYBPC3, MYH6, MYH7, MYL2, MYL3, MYLK2, MYOZ2, MYPN, NEBL, NEXN, NPPA, PDLIM3, PKP2, PLN, PRDM16, PRKAG2, RAF1, RBM20, RYR2, SCN10A, SCN1B, SCN2B, SCN3B, SCN4B, SCN5A, SDHA, SGCD, SMAD4, SNTA1, TAZ, TCAP, TGFB3, TMEM43, TMPO, TNNC1, TNNI3, TNNT2, TPM1, TRDN, TRPM4, TTN, TTR, VCL |
| **Module 3: Coagulation** | Thrombophilia, hemophilia  14 genes | F13A1, F2, F5, F9, HRG, PLAT, PLI, PROC, PROS1, SERPINC1, SERPIND1, THBD, VWD1, VWF |
| **Module 4: Storage disorders** | Hemochromatosis, Wilson’s disease  6 genes | ATP7B, ATP7A, HFE, HJV, SLC40A1, TFR2 |
| **Module 5: Hypercholesterolemia** | 10 genes | APOA2, ITIH4, GHR, GSBS, EPHX2, ABCA1, LDLR, LDLRAP1, APOB, PCSK9 |
| **Module 6: Glaucoma** | 4 genes | MYOC, CYP1B1, CYP1B1, OPTN |
| **Module 7: Pharmacogenomics** | e.g. Warfarin dosage, tolerance to succinylcholine  Contains PGX34 - the 34 ADME genes that are best studied  38 genes | ABCB1, ABCC2, ABCG2, CACNA1S, CYP1A1, CYP1A2, CYP2A6, CYP2B6, CYP2C19, CYP2C8, CYP2C9, CYP2D6, CYP2E1, CYP3A4, CYP3A5, DPYD, EPHX1, GSTM1, GSTP1, GSTT1, HLA-B, NAT1, NAT2, RYR1, SLC15A2, SLC22A1, SLC22A2, SLC22A6, SLCO1B1, SLCO1B3, STAC3, SULT1A1, TPMT, UGT1A1, UGT2B15, UGT2B17, UGT2B7, VKORC1 |

| **Supplementary Table 2. Number of positive findings in the genetic testing. Clinical relevance information based on the documentation of the genetic test and gene databases. n=241** | | |
| --- | --- | --- |
|  |  |  |
| **Tested module** | **Number of participants with positive finding per category (%, n=241)** | **Number of clinically relevant risk genes and diseases linked to genetic variants** |
| **Participants with any positive finding without pharmacogenomics** | 60 (24.9%) |  |
| **Malignancies** | 22 (9%) | \| **number** \| **Gene name** \| **Clinical significance** \| \| --- \| --- \| --- \| \| 5 \| BRCA1 \| Breast and ovarian cancers \| \| 4 \| SPINK1 \| Possible clinical relevance in pancreatitis \| \| 3 \| MUTYH \| Possible clinical relevance in familial adenomatous polyposis and pilomatrixoma \| \| 3 \| PALB2 \| Pancreatic cancer and Fanconi anemia \| \| 2 \| LZTR1 \| Possible clinical relevance in schwannomatosis and Noonan syndrome \| \| 1 \| BRCA2 \| Fanconi anemia and breast cancer \| \| 1 \| MLH1 \| Colorectal cancer, hereditary nonpolyposis, Type 2 and mismatch repair cancer syndrome \| \| 1 \| NBN \| Nijmegen breakage syndrome and aplastic anemia \| \| 1 \| TP53 \| Li-Fraumeni syndrome and osteogenic sarcoma \| \| 1 \| SDHAF2 \| Paragangliomas and hereditary paraganglioma-pheochromocytoma syndromes \| |
| **Cardiology** | 6 (2%) | \| 2 \| MYBPC3 \| Familial hypertrophic cardiomyopathy and dilated cardiomyopathy \| \| --- \| --- \| --- \| \| 1 \| MFAP5 \| Possible clinical relevance in aneurysm formation \| \| 1 \| SCN5A \| Brugada/longQT syndrome \| |
| **Coagulation** | 34 (14%) | \| 20 \| F5 \| Factor V deficiency and thrombophilia due to activated protein C resistance \| \| --- \| --- \| --- \| \| 8 \| F2 \| Thrombophilia, prothrombin deficiency, stroke \| \| 1 \| FBN1 \| Possible clinical relevance in stiff skin syndrome and Marfan lipodystrophy syndrome \| \| 1 \| VWF \| Hemophilia, Von Willebrand disease \| |
| **Storage disorders** | 29 (12%) | \| 6 (only compound heterozygote) \| HFE \| Hemochromatosis \| \| --- \| --- \| --- \| |
| **Cholesterol** | 5 (2%) | \| 4 \| LDLR \| Hypercholesterolemia \| \| --- \| --- \| --- \| |
| **Glaucoma** | 4 (0%) |  |
| **Pharmacogenomics** | 238 (98%) |  |

| **Supplementary Table 3. Definition of preferable behavioral change regarding changes in nutrition per item in the LEI questionnaire.** | | | |
| --- | --- | --- | --- |
|  |  | | |
| **Item group** | **increase in items for the evaluation FU>BL to be preferred (more/higher after consultation is better)** | **decrease in items for the evaluation FU>BL to be preferred (lower/fewer after consultation is better)** | **no rating of probability, indifferent** |
|  | How often do you have lunch? |  |  |
| **Beverages** | Tea /Coffee | Milk, cocoa, coffee with milk |  |
|  | Water | Fruit juice |  |
|  |  | Sweetened drinks |  |
|  |  | Alcoholic beverages |  |
| **Bread, bread rolls with** | Fish, seafood | Frequency of Bread, bread rolls |  |
|  |  | Butter, margarine, mayonnaise |  |
|  |  | Egg |  |
|  |  | Meat |  |
|  |  | Cheese, cream cheese |  |
|  |  | Sausage, ham, salami |  |
| **Warm meal, consisting of** | Side salad | Egg dish |  |
|  | Fish, seafood | Beef, pork, lamb, other red meat |  |
|  | Meat | Chicken, turkey |  |
|  | Vegetables | Fried food |  |
|  | Legumes | Potatoes, rice, pasta |  |
|  | Vegetarian dish | Pizza |  |
|  |  | Soup |  |
|  |  | Sausages |  |
| **Cold meal, consisting of** | Fish, seafood | Eggs |  |
|  | Legumes | Meat |  |
|  | Salad plate | Beef, pork, lamb, other red meat |  |
|  | Fresh fruits | Chicken, turkey |  |
|  | Yoghurt, curd cheese, cottage cheese | Sausages |  |
|  | Muesli, cereal flakes (oats, spelt, etc.) | Salads made from potatoes, rice, pasta | |
|  | Nuts, salt almonds | Salty "snack food“ |  |
|  |  | Chocolate, chocolate products |  |
|  |  | Sweet pastries |  |
|  |  | Sweet "snack food" |  |
|  |  | How often do you eat a fast food menu for lunch? | |
| **Where do you have your lunch?** | at home | directly at my workplace (incl. vehicle) | on the way in a bar, restaurant, café etc. |
|  |  |  | in a company canteen |
|  |  |  | in our company kitchen |
| **Where does the food/drink for lunch come from?** | from home |  | bought on the way |
|  |  |  | from where I eat (bar, café, canteen) |
|  | Do you do something on the side at lunch ? | keep working, do other work | reading, watching TV |
|  |  | making phone calls | Listening to the radio, listening to music |

| **Supplementary Table 4. All qualitative commentaries of different study participants regarding lifestyle changes in response to the results of the genetic test.** |
| --- |
| I pay more attention to reducing fatty meals and sweets. |
| reduction in calorie intake. |
| more fruit, vegetables. |
| more salads and vegetables. |
| lots of fruit and vegetables, little sugar and carbohydrates, a lot of drinking: 2.5-4L water. |
| try to do more sports. |
| less carbohydrates, less alcohol. |
| more conscious, metabolic balance. |
| less snacks, more fruit, more exercise. |
| consumption of sugar, carbohydrates (further) reduced. |
| regular jogging. |
| fewer meat dishes, more vegetarian. |
| less calories, more fruit, less candy. |
| conscious eating. |
| less carbohydrates, more vegetables. |
| more sport, with bicycle daily. In company, no chocolate (...) muesli for breakfast with fruit instead of bread and Nutella. |
| in the cafeteria often vegetarian. |
| less alcohol, more vegetarian. |
| less carbohydrates. |
| less calories, more vegetables. |
| more conscious, rarely sausage and meat. |
| more fruit and vegetables, max. 3x a week meat. |
| more often vegetarian, more varied. |
| more water, try to eat less sugar, some fruit. |
| more vegetables and fruit, less alcohol. |
| try eating less in the evening, more fruit and vegetables. |
| more vegetables and less candy. |

| **Supplementary Figure 1. Subgroup comparison per module of differences in outcome measures between follow-up and baseline** |
| --- |
| **** |
| Dots = estimated mean difference after adjusting for age, gender, and body mass index, whiskers = 95% confidence interval, clipped when exceeding the displayed range, X-axis: the estimated mean difference between follow-up and baseline (FU-BL) is given on the y-axis, except for IPAQ MET-min, where we consider the difference of logarithmized values.  Abbreviations: BL = baseline, FU = follow-up, h = hour, IPAQ = International Physical Activity Questionnaire, MET-min = metabolic equivalent of task per minute, min = minute, n = number, p = raw p-values, SF36msc = mental health subscale of the Short-Form Health Survey, SF36psc = physical health subscale of the Short-Form Health Survey. Graph was created with R [1]. |

| **Supplementary Figure 2. Change in nutrition (at lunchtime) per questionnaire item of the LEI score for the entire cohort and subgroups.** |
| --- |
|  |
| Raw p-values in columns, underlined and bold when <0.05, n= number of cases, highlighted green = difference towards direction of desired behavioral changes, highlighted red = difference towards direction of undesired behavioral change, highlighted gray = no rating, indifferent. Graph was created with R [1]. |

**References**

1. R Core Team. R: A language and environment for statistical computing. Vienna, Austria: R Foundation for Statistical Computing; 2020.
