# Supplementary material for: Physician-guided, hybrid genetic testing exerts promising effects on health-related behavior without compromising quality of life
Source: Sci Rep. 2021 Apr 19;11:8494. doi: 10.1038/s41598-021-87821-8 (PMC8055666; doi:10.1038/s41598-021-87821-8)
Supplement: Supplementary file 2 — Supplementary Figure 1. [file 41598_2021_87821_MOESM2_ESM.pdf]

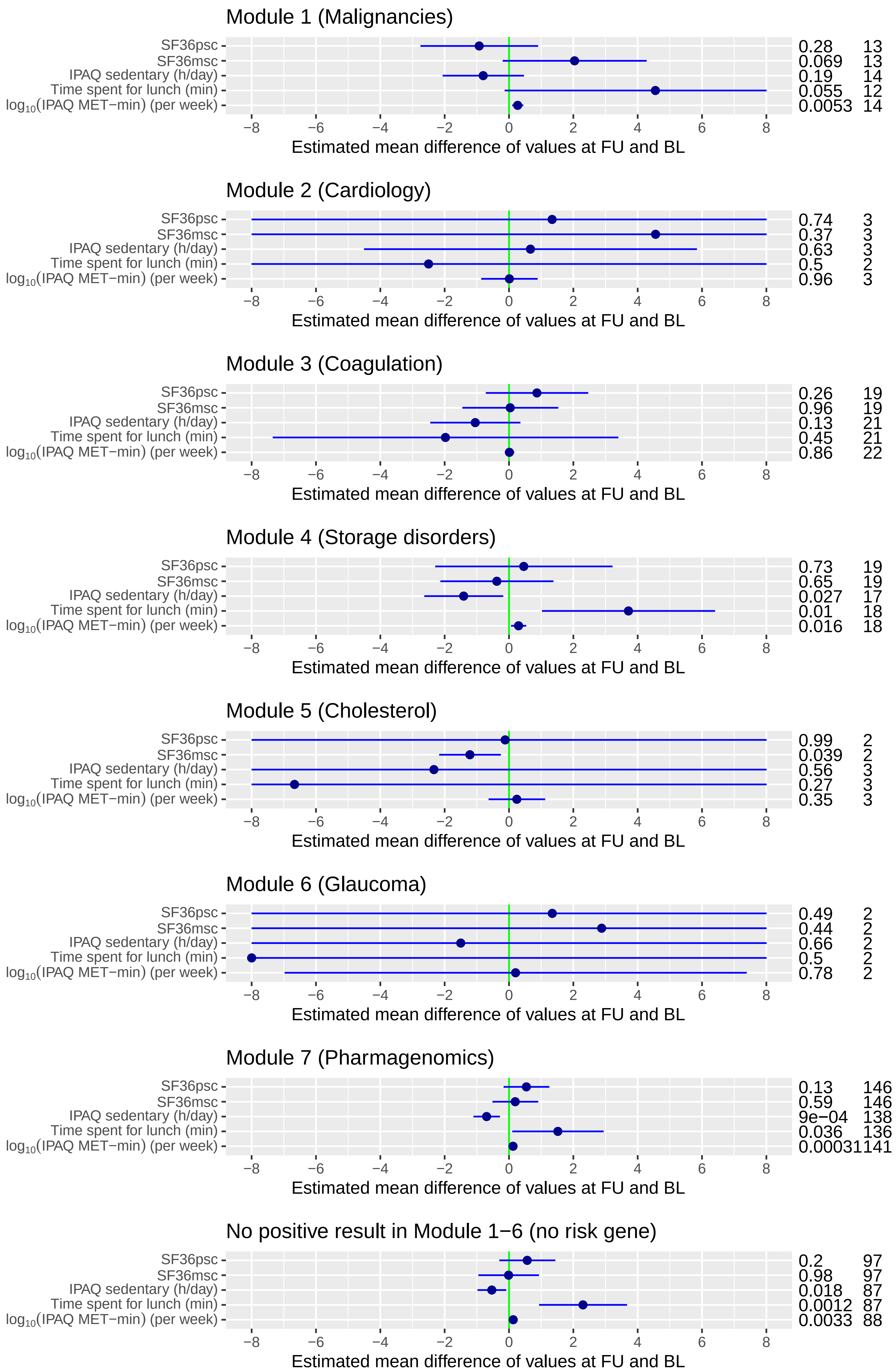
Module 1 (Malignancies)
SF36psc
SF36msc
IPAQ sedentary (h/day)
Time spent for lunch (min)
log$_{10}$(IPAQ MET−min) (per week)
0.28 13
0.069 13
0.19 14
0.055 12
0.0053 14
−8 −6 −4 −2 0 2 4 6 8
Estimated mean difference of values at FU and BL
Module 2 (Cardiology)
SF36psc
SF36msc
IPAQ sedentary (h/day)
Time spent for lunch (min)
log$_{10}$(IPAQ MET−min) (per week)
0.74 3
0.37 3
0.63 3
0.5 2
0.96 3
−8 −6 −4 −2 0 2 4 6 8
Estimated mean difference of values at FU and BL
Module 3 (Coagulation)
SF36psc
SF36msc
IPAQ sedentary (h/day)
Time spent for lunch (min)
log$_{10}$(IPAQ MET−min) (per week)
0.26 19
0.96 19
0.13 21
0.45 21
0.86 22
−8 −6 −4 −2 0 2 4 6 8
Estimated mean difference of values at FU and BL
Module 4 (Storage disorders)
SF36psc
SF36msc
IPAQ sedentary (h/day)
Time spent for lunch (min)
log$_{10}$(IPAQ MET−min) (per week)
0.73 19
0.65 19
0.027 17
0.01 18
0.016 18
−8 −6 −4 −2 0 2 4 6 8
Estimated mean difference of values at FU and BL
Module 5 (Cholesterol)
SF36psc
SF36msc
IPAQ sedentary (h/day)
Time spent for lunch (min)
log$_{10}$(IPAQ MET−min) (per week)
0.99 2
0.039 2
0.56 3
0.27 3
0.35 3
−8 −6 −4 −2 0 2 4 6 8
Estimated mean difference of values at FU and BL
Module 6 (Glaucoma)
SF36psc
SF36msc
IPAQ sedentary (h/day)
Time spent for lunch (min)
log$_{10}$(IPAQ MET−min) (per week)
0.49 2
0.44 2
0.66 2
0.5 2
0.78 2
−8 −6 −4 −2 0 2 4 6 8
Estimated mean difference of values at FU and BL
Module 7 (Pharmagenomics)
SF36psc
SF36msc
IPAQ sedentary (h/day)
Time spent for lunch (min)
log$_{10}$(IPAQ MET−min) (per week)
0.13 146
0.59 146
9e−04 138
0.036 136
0.00031141
−8 −6 −4 −2 0 2 4 6 8
Estimated mean difference of values at FU and BL
No positive result in Module 1−6 (no risk gene)
SF36psc
SF36msc
IPAQ sedentary (h/day)
Time spent for lunch (min)
log$_{10}$(IPAQ MET−min) (per week)
0.2 97
0.98 97
0.018 87
0.0012 87
0.0033 88
−8 −6 −4 −2 0 2 4 6 8
Estimated mean difference of values at FU and BL
