# Supplementary figures and images for: Physician-guided, hybrid genetic testing exerts promising effects on health-related behavior without compromising quality of life
Source: Sci Rep. 2021 Apr 19;11:8494. doi: 10.1038/s41598-021-87821-8 (PMC8055666; doi:10.1038/s41598-021-87821-8)

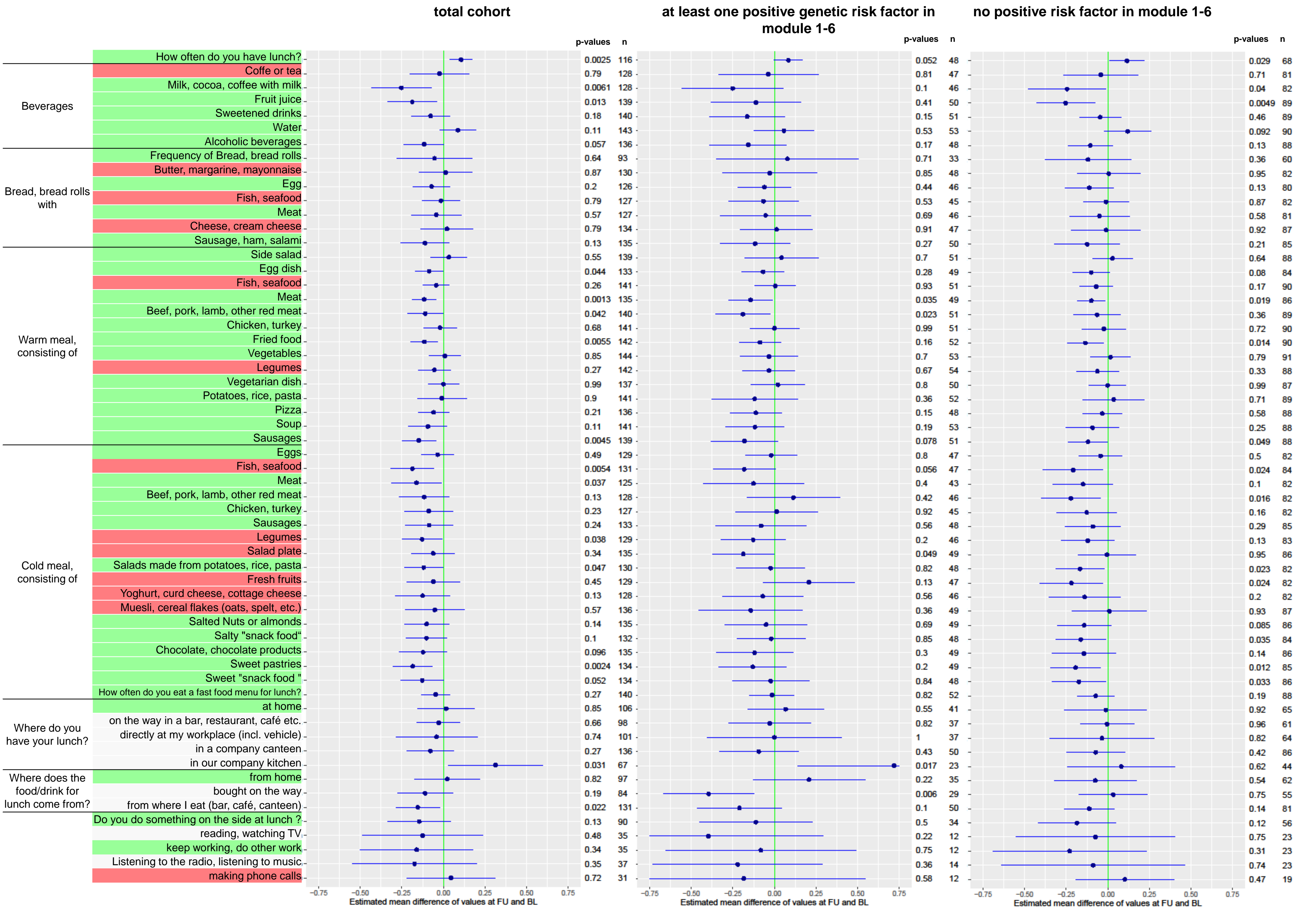

Supplement: Supplementary file 3 — Supplementary Figure 2. [file 41598_2021_87821_MOESM3_ESM.pdf]
